# Time trend analysis (2009-2016) of antimicrobial susceptibility in *Neisseria gonorrhoeae* isolated in Italy following the introduction of the combined antimicrobial therapy

**DOI:** 10.1371/journal.pone.0189484

**Published:** 2017-12-14

**Authors:** Paola Stefanelli, Maria Fenicia Vescio, Maria Paola Landini, Ivano Dal Conte, Alberto Matteelli, Antonio Cristaudo, Marina Gaino, Marco Cusini, Anna Maria Barbui, Antonella Mencacci, Rosella De Nittis, Valeria Ghisetti, Elena Stroppiana, Anna Carannante, for The Neisseria gonorrhoeae antimicrobials resistant Study Group

**Affiliations:** 1 Department Infectious Diseases, Istituto Superiore di Sanità, Rome, Italy; 2 Unit of Clinical Microbiology, University Hospital S.Orsola-Malpighi, Bologna, Italy; 3 STI Clinic, Dept. of Infectious Diseases, Amedeo di Savoia Hospital, Turin, Italy; 4 Clinic of Infectious and Tropical Diseases, University of Brescia, Brescia, Italy; 5 San Gallicano Dermatologic Institute, IRCCS, Rome, Italy; 6 Microbiology and Virology Laboratory, Santa Chiara Hospital, Trento, Italy; 7 Foundation IRCCS Ca’ Granda ‘Ospedale Maggiore Policlinico Milano’, Milan, Italy; 8 Microbiology and Virology Laboratory, Molinette Hospital, Turin, Italy; 9 Medical Microbiology Section, Dept. of Medicine, University of Perugia, Perugia, Italy; 10 Department of Clinical Pathology, ‘Azienda Ospedaliero-Universitaria OORR’, Foggia, Italy; 11 Laboratory of Microbiology and Virology, Amedeo di Savoia Hospital, Turin, Italy; 12 Dermatologic Clinic, ‘A.O.U. Città della Salute e della Scienza’, Turin, Italy; Emory University School of Medicine, UNITED STATES

## Abstract

**Introduction:**

*Neisseria gonorrhoeae* (NG) antimicrobial susceptibility trends to azithromycin, cefixime and ceftriaxone were analyzed, from 2009 to 2016, to monitor changing antimicrobial susceptibility concomitant with the change in prescribing practice in 2012 from cefixime, or ceftriaxone, to ceftriaxone plus azithromycin. Patient characteristics predictive to be infected by antibiotic resistant *N*. *gonorrhoeae* were estimated. Finally, the protocol for the treatment of gonorrhoea, in comparison with the international guidelines, was also evaluated.

**Materials and methods:**

Data on NG antimicrobial resistance were obtained from a network of sexually transmitted diseases clinics and other laboratories in 12 cities in Italy. We tested the 1,433 gonococci for antimicrobial susceptibility to azithromycin, cefixime and ceftriaxone using a gradient diffusion method. Logistic-regression methods with cluster robust standard errors were used to investigate the association of resistance categories with demographic and clinical patient characteristics and to assess changes in prescribing practices. To minimize bias due to missing data, all statistical models were fitted to data with forty rounds of multiple imputation, using chained equations.

**Results:**

The percentage of isolates resistant to cefixime was 17.10% in 2009 and declined up to 1.39% in 2016; at the same time, those resistant to azithromycin was 23.68% in 2009 and 3.00% in 2012. Starting from 2013, azithromycin resistant gonococci tended to increase up to 7.44% in 2016. No ceftriaxone resistant isolates were observed. By multivariate analysis, the men who have sex with women (MSW) and women had a proportional adjusted OR of resistance of 1.25 (95%CI: 0.90; 1.73) and 1.67 (95%CI: 1.16; 2.40), respectively, in comparison with men who have sex with men (MSM). An aOR of resistance of 0.48 (95%CI: 0.21; 1.12) among NG isolated in the pharynx, compared with those isolated in genital sites, was calculated. The proportional aOR of resistance was 0.58 (95%CI: 0.38; 0.89) for presence *vs* absence of co-infection and 2.00 (95%CI: 1.36; 2.96) for past history *vs* no history of gonorrhoea.Finally, at least for the period 2013–2016, the older, subjects with anorectal or pharyngeal gonorrhoea infection, subjects with a co-infection, subjects with a previous gonorrhoea infection were not always correctly treated.

**Conclusions:**

Overall, our findings suggest the shifts in *N*. *gonorrhoeae* susceptibility to cefixime and azithromycin in the time frame period. First of all, the increasing rate of azithromycin resistance in 2015–2016 in NG isolated in the country need to be monitor in the future. Finally, extensive information on treatment regimens may be useful to asses treatment adherence particularly for the older subjects, subjects with an anorectal or pharyngeal infection, subjects with a co-infection and subjects with a previous history of gonorrhoea. Gonorrhoea treatment strategy should be based on the evidence obtained by the local antimicrobial surveillance system and data about treatment failures.

## Introduction

The rapid and global spread of antimicrobial resistance in *Neisseria gonorrhoeae* (NG) is unprecedented. Several clinical reports of monotherapy treatment failure have been published in the past [1–7], due to isolates with high cefixime (CFM) and ceftriaxone (CRO) minimum inhibitory concentration (MIC), >0.125 mg/L. Therefore, the 2009 European treatment guidelines [8], indicating third-generation extended-spectrum cephalosporins (ESCs), CRO or CFM, as a monotherapy, was updated in 2012 introducing the use of combined therapy with azithromycin (AZM) and ESCs [9]. In particular, an intra-muscular (*i*.*m*.) 500 mg single dose of CRO combined with 2g of oral AZM is recommended for uncomplicated NG infection of the urethra, cervix and rectum [9]. A single dose of CFM 400 mg could be used as alternative treatment together with AZM, if CRO *i*.*m*. is not available or unacceptable to the patients [9].

Gonococci resistant to ESCs and AZM have been described in Europe and worldwide [3, 4, 6, 10–12]. The European Gonococcal Antimicrobial Surveillance Programme (Euro-GASP) reported a decrease of resistance to CFM (MIC >0.125 mg/L) from 4.7% in 2013 to 2.0% in 2014 [13]. Additionally, a mean of AZM resistance (MIC >0.5 mg/L) of 7.9% in 2014 has been described [13].

The percentages of CFM and AZM resistance were similar in 2015, compared to 2014: 1.7% and 7.1%, respectively [14].

In Italy, in 2015, 650 gonorrhoea cases were reported [15] and the rate of gonococci with resistance to CFM and AZM decreased from 11% in 2008 to 3.3% in 2012, and from 14% in 2007 to 2.2% in 2012, respectively [16]; moreover, high levels of resistance to AZM (MICs of 128 or 256 mg/L) was also reported in 2008 [17]. At that time, AZM was used for treatment of *Chlamydia trachomatis* infection only [8, 9].

Recent studies reported a higher frequency of infections due to resistant gonococci among men who have sex with men (MSM) and a greater frequency of infection due to NG belonging to the same clone, the genogroup (G) 1407, which is associated with a decreased susceptibility to CFM [4]. It has also been hypothesized that some mechanisms that lead to antimicrobial resistance may be more efficient among NG isolated in MSM [18, 19] and, for this reason, in the past it was also discussed the possibility of having differential treatment guidelines referring to sexual orientation [20, 21].

The main goal of this study was to investigate the trend of antimicrobial susceptibility to AZM, CFM and CRO, from 2009 to 2016, after the introduction of the 2012 treatment guidelines.

Secondary aims were to identify patient characteristics predictive for a resistant isolate infection and to compare the adherence between prescribed clinical practice to the current guidelines.

## Results

### Antimicrobial susceptibility and resistance

A total of 1,433 culture positive gonorrhoea cases with available data about antimicrobial susceptibility (for AZM, CFM and CRO) and therapy information were collected from 2009 to 2016 (666 isolates were collected between 2009 and 2012 and 767 between 2013 and 2016).

Overall, 73 azithromycin resistant (AZM-R), 35 cefixime resistant (CFM-R) and 12 azithromycin and cefixime resistant (AZM-R/CFM-R) gonococci were identified. The percentage of susceptibility to CFM was 97% among MSW and 99% among MSM. All isolates were susceptible to CRO.

**Fig 1:**
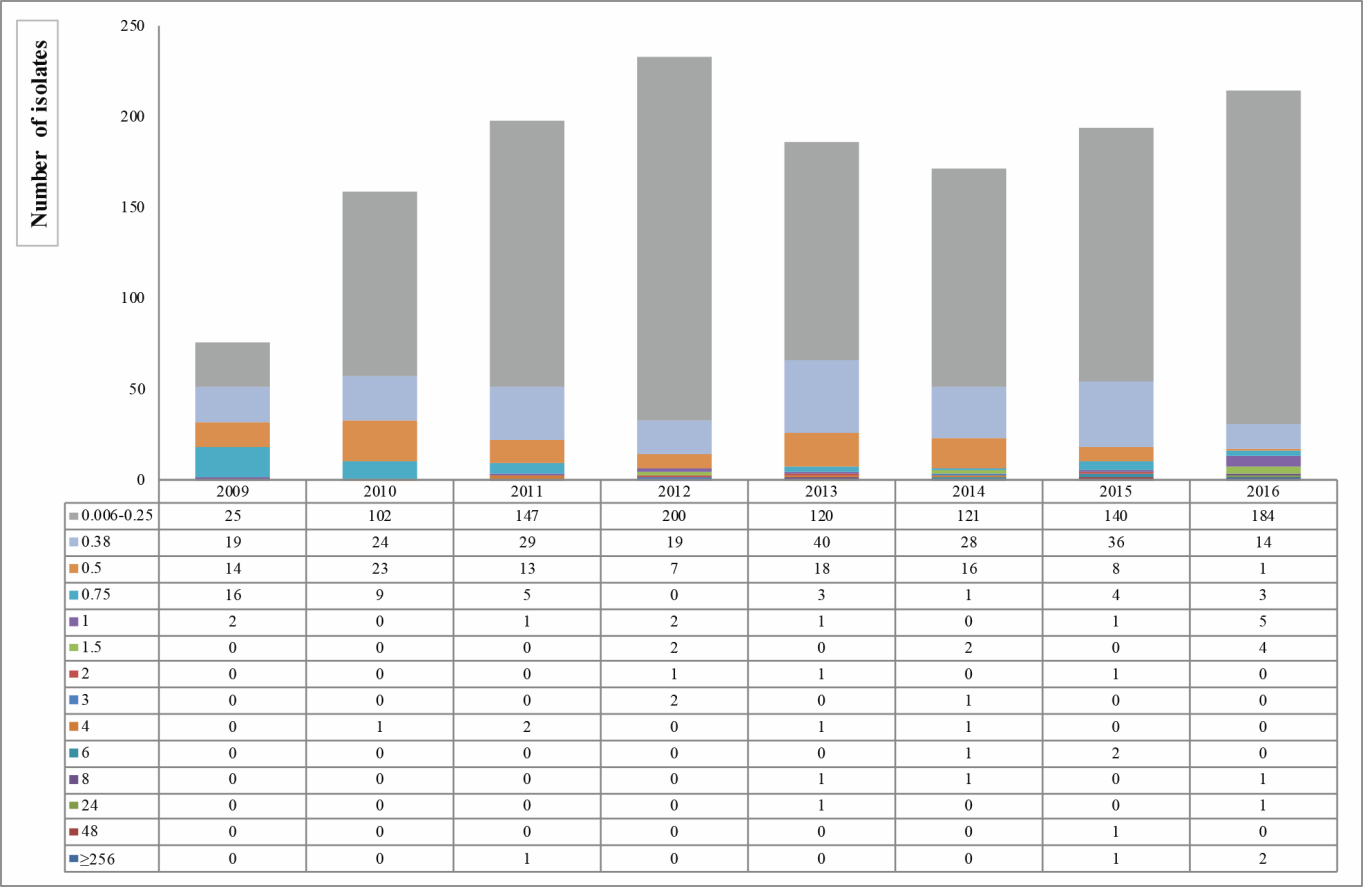
Distribution of azithromycin MIC values (mg/L) on 1,433 *Neisseria gonorrhoeae* isolates, from 2009 to 2016 in Italy.

Fig 1 shows the distribution of AZM MIC values of 1,433 gonococci, during the years. From 2009 to 2016, the majority of isolates were susceptible: 72.50% (1,039/1,433) with a MIC range of 0.006–0.25 mg/L. The MIC_50_ and MIC_90_ were 0.19 mg/L and 0.5 mg/L, respectively.

The 21.56% of isolates were intermediate to AZM (AZM-I; MIC range of 0.38–0.5 mg/L), in particular: 209 gonococci had a MIC value of 0.38 mg/L and 100 a MIC value of 0.5 mg/L. The 5.93% (N = 85/1,433) were resistant and a total of 4 isolates with high level of resistance, with a MIC value of ≥ 256 mg/L, were identified: one isolate in each of the years between 2011 and 2015 and two in 2016.

**Fig 2:**
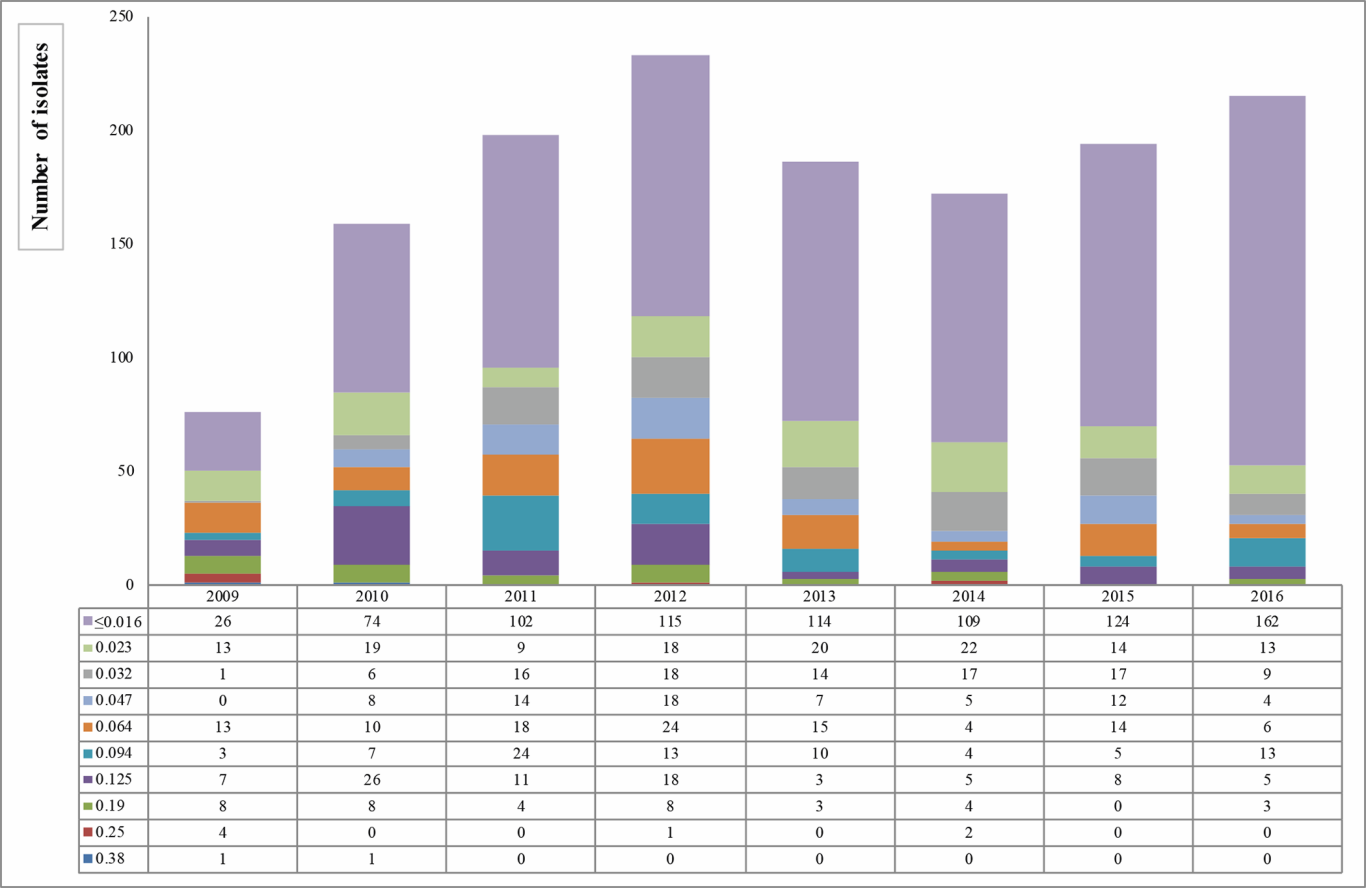
Distribution of cefixime MIC values (mg/L) on 1,433 *Neisseria gonorrhoeae* isolates, from 2009 to 2016 in Italy.

Fig 2 shows the distribution of CFM MIC values between 2009 and 2016. The majority of isolates were susceptible, with MIC values lower than the breakpoint of 0.125 mg/L (Fig 2). In particular, 57.64% (826/1,433) of them showed a MIC value of ≤ 0.016 mg/L followed by the 8.93% (128/1,433) with a value of 0.023 mg/L. The percentage of resistant gonococci (MIC range of 0.19–0.38 mg/L) decreased from 2009 (N = 13, 17.10%) to 2016 (N = 3, 1.40%).

**Fig 3:**
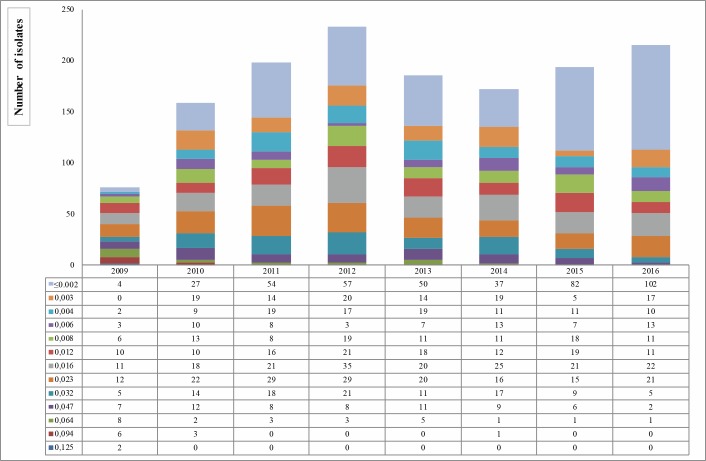
Distribution of ceftriaxone MIC values (mg/L) on 1,433 *Neisseria gonorrhoeae* isolates, from 2009 to 2016 in Italy.

Fig 3 shows the distribution of CRO MIC values between 2009 and 2016. No resistant gonococci were detected. The 28.82% of the isolates (413/1,433) were fully susceptible with a MIC value of ≤ 0.002 mg/L. A total of 12 isolates with a MIC value near to the breakpoint of 0.125 mg/L (0.094–0.125 mg/L) were found: 8 in 2009, 3 in 2010 and 1 in 2014.

**Fig 4:**
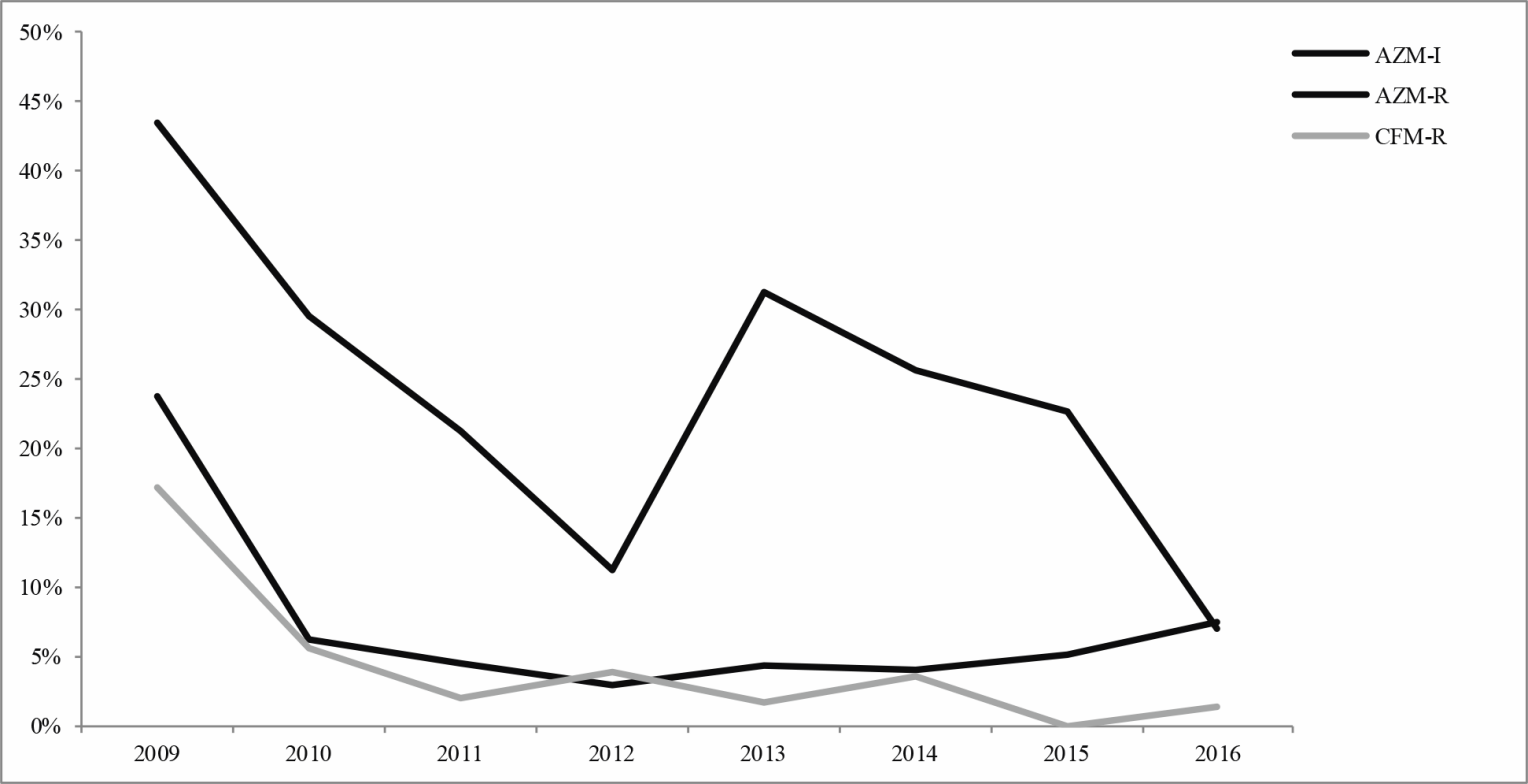
Percentage of gonococci intermediate and resistant to azithromycin and resistant to cefixime per years, 2009–2016, in Italy. In the 2012 the new recommendations for the antimicrobial gonorrhoea treatment were introduced in Europe [9].

Time trends of AZM-R, AZM-I and of CFM-R were reported in Fig 4. The percentage of AZM-R gonococci seemed to decrease since 2009 (23.68%) and until 2012 (3.00%), time when the new recommendations on the use of dual therapy were introduced in Europe. Since 2013, the rate of AZM-R tended to increase reaching 7.44% in 2016. The number of AZM-I gonococci decreased from 2009 (43.42%) to 2012 (11.15%), increased with a peak in 2013 (31.18%) and decreased afterwards reaching the lowest value in 2016 (6.97%).

Also for CFM-R a decrease was observed from 2009 (17.10%) to 2011 (2.00%). After 2011, the percentages of CFM-R gonococci have remained fairly stable until 2014 (3.48%). Then they decreased in 2015 (when no gonococci CFM-R were detected) and in 2016 (1.39%).

### Association between patient characteristics and resistance

Table 1 summarizes the demographic and clinical characteristics of the 1,433 culture positive gonorrhoea isolates by antimicrobial resistance categories for AZM and CFM. The 96.86% of gonococci were isolated from males (1,388/1,433). Overall, the median age was 31 years (range 16–86 years): the 23.80% of gonococci were isolated from subjects aged 25 years or younger and the 76.20% from those older than 25 years. A total of 878 (61.44%) isolates were from MSM, 506 (35.41%) from men who have sex with women (MSW) and 45 (3.15%) from women. The majority of gonococci were isolated from genital sites (86.44%). Isolates collected from HIV positive patients, patients with a co-infection and whom with a previous gonorrhoea infection were 13.19% (189/1,433), 13.35% (149/1,116) and 16.60% (199/1,199), respectively. Among co-infected patients, the majority (121/149; 81.21%) had chlamydial infection. Most gonococci came from Italian patients (1,207/1,433; 84.35%), the 5.94% (85/1,433) from East Europeans, the 5.03% (72/1,433) from Africans, 3.28% (47/1,433) from Latin Americans and 1.40% (20/1,433) from Asians.

**Table 1:** Results from the NG antimicrobial resistance surveillance in Italy (2009–2016): Demographic and clinical characteristics of 1,433 subjects from which samples were collected by antibiotic resistance categories.

	All		AZM-S/CFM-S		AZM-I/CFM-S		AZM-R or CFM-R		AZM-I/CFM-R		AZM-R/CFM-R-	
	N	(%)	N	(%)	N	(%)	N	(%)	N	(%)	N	(%)
**Year of infection**												
*2009–2012*	666	(46.48)	461	(69.22)	135	(20.27)	48	(7.21)	13	(1.95)	9	(1.35)
*2013–2016*	767	(53.52)	559	(72.88)	158	(20.60)	44	(5.74)	3	(0.39)	3	(0.39)
**Sexual orientation and gender**												
*MSM*	878	(61.44)	637	(72.55)	174	(19.82)	51	(5.81)	9	(1.03)	7	(0.80)
*MSW*	506	(35.41)	349	(68.97)	109	(21.54)	37	(7.31)	7	(1.38)	4	(0.79)
*Women*	45	(3.15)	31	(68.89)	9	(20.00)	4	(8.89)	0	(0.00)	1	(2.22)
**Age (years)**												
*< = 25*	341	(23.80)	252	(73.90)	57	(16.72)	29	(8.50)	1	(0.29)	2	(0.59)
*>25*	1092	(76.20)	768	(70.33)	236	(21.61)	63	(5.77)	15	(1.37)	10	(0.92)
**Citizenship**												
*Italian*	1207	(84.35)	855	(70.84)	246	(20.38)	84	(6.96)	11	(0.91)	11	(0.91)
*Asian*	20	(1.40)	14	(70.00)	6	(30.00)	0	(0.00)	0	(0.00)	0	(0.00)
*Est European*	85	(5.94)	68	(80.00)	14	(16.47)	2	(2.35)	1	(1.18)	0	(0.00)
*Latin American*	47	(3.28)	36	(76.60)	9	(19.15)	1	(2.13)	0	(0.00)	1	(2.13)
*African*	72	(5.03)	45	(62.50)	18	(25.00)	5	(6.94)	4	(5.56)	0	(0.00)
**Site of infection**												
*Genital*	1230	(86.44)	875	(71.14)	255	(20.73)	81	(6.59)	11	(0.89)	8	(0.65)
*Anorectal*	168	(11.81)	119	(70.83)	32	(19.05)	10	(5.95)	4	(2.38)	3	(1.79)
*Pharingeal*	25	(1.76)	20	(80.00)	5	(20.00)	0	(0.00)	0	(0.00)	0	(0.00)
**HIV status**												
*Negative*	1244	(86.81)	890	(71.54)	247	(19.86)	84	(6.75)	12	(0.96)	11	(0.88)
*Positive*	189	(13.19)	130	(68.78)	46	(24.34)	8	(4.23)	4	(2.12)	1	(0.53)
**Co-infection**^a^												
*No*	967	(86.65)	711	(73.53)	189	(19.54)	56	(5.79)	6	(0.62)	5	(0.52)
*Yes*	149	(13.35)	122	(81.88)	22	(14.77)	5	(3.36)	0	(0.00)	0	(0.00)
**Previously diagnosed**^b^												
*No*	1000	(83.40)	749	(74.90)	189	(18.90)	51	(5.10)	6	(0.60)	5	(0.50)
*Yes*	199	(16.60)	134	(67.34)	41	(20.60)	22	(11.06)	1	(0.50)	1	(0.50)

AZM-S/CFM-S: “susceptible to AZM and CFM”, AZM-I/CFM-S: “intermediate to AZM and susceptible to CFM”, AZM-R or CFM-R: “resistant to AZM and susceptible to CFM or resistant to CFM and susceptible to AZM”, AZM-I/CFM-R: “susceptible or intermediate to AZM and resistant to CFM”, AZM-R/CFM-R: “resistant to AZM and CFM”. Data do not sum to total because of missing values.

^a^ 317 data were missing

^b^ 234 data were missing

Univariate and multivariate analyses were reported in Table 2. Lower levels of resistance were found for isolates collected in 2013–2016 in comparison with those collected in 2009–2012 with an OR of higher (to lower) resistance of 0.82 (95% CI: 0.68; 0.97) [meaning that for any resistance category, isolates collected after 2012 had a 18% lower probability of being resistant than before]. MSW isolates had proportional OR of resistance of 1.21 (95%CI: 0.97;1.49) [*i*.*e*. compared to MSM isolates, the odds of having gonococci resistant or intermediate to at least one antimicrobial (AZM-I/CFM-S, AZM-R/CFM-S or AZM-S/CFM-R, AZM-I/CFM-R, AZM-R/CFM-R) *vs* AZM-S/CFM-S was 21% higher for MSW ones. The same increase was found between the odds of having gonococci resistant to at least one antimicrobial (AZM-R/CFM-S or AZM-S/CFM-R, AZM-I/CFM-R, AZM-R/CFM-R) *vs* all the other combined categories (AZM-S/CFM-S, AZM-I/CFM-S), between AZM-I/CFM-R and AZM-R/CFM-R *vs* all the other combined categories (AZM-I/CFM-S, AZM-R/CFM-S or AZM-S/CFM-R, AZM-S/CFM-S) and between AZM-R/CFM-R *vs* all other combined categories (AZM-I/CFM-R, AZM-R/CFM-S or AZM-S/CFM-R, AZM-I/CFM-S, AZM-S/CFM-S)]. Women had a OR of 1.24 (95%CI: 0.88;1.75) for resistance. Isolates from older subjects were more likely to be resistant (OR: 1.14; 95%CI: 0.95; 1.37) than those collected from younger subjects. Apart for Africans, gonococci isolated from subjects of other nationalities were more likely to be resistant compared with those collected from Italians. Compared to isolates retrieved from the genital site, those from the pharyngeal and anorectal sites were 39% less (OR:0.61; 95%CI: 0.27; 1.38) and 5% more (OR:1.05; 95%CI: 0.94; 1.17) likely to be resistant, respectively. The probability of being infected by resistant isolates was lower among the co-infected people than not co-infected (OR: 0.65; 95%CI: 0.44; 0.97), quite the opposite was found for isolates collected from patients with a previous history of gonorrhoea (OR: 1.81; 95%CI: 1.24; 2.66). No evidence of association between resistance severity and HIV status was found.

**Table 2:** Results from the NG antimicrobial resistance surveillance in Italy (2009–2016): ORs and 95%CI from univariate, and aORs (adjusted OR) and 95%CI from a multivariate ordered logistic model for resistance categories with cluster adjusted standard errors.

	N	(%)	OR^c^	(95%CI)	p	aOR^c^	(95%CI)	p
**Year of infection**					0.028	NI		
*2009–2012*	666	(46.48)	1					
*2013–2016*	767	(53.52)	0.82	(0.68; 0.97)				
**Sexual orientation and gender**					0.181			
*MSM*	878	(61.44)	1			1		
*MSW*	506	(35.41)	1.21	(0.97; 1.49)		1.25	(0.90; 1.73)	0.178
*Women*	45	(3.15)	1.24	(0.88; 1.75)		1.67	(1.16; 2.40)	0.005
**Age (years)**					0.093			
*< = 25*	341	(23.80)	1			1		
*>25*	1092	(76.20)	1.14	(0.95; 1.37)		1.14	(0.93; 1.33)	0.080
**Citizenship**					<0.001			
*Italian*	1207	(84.35)	1			1		
*Asian*	20	(1.40)	0.93	(0.73; 1.18)		0.81	(0.68; 0.98)	0.027
*Est European*	85	(5.94)	0.60	(0.35; 1.02)		0.52	(0.31; 0.87)	0.013
*Latin American*	47	(3.28)	0.73	(0.46; 1.15)		0.72	(0.46; 1.12)	0.139
*African*	72	(5.03)	1.49	(1.05; 2.13)		1.38	(0.90; 2.11)	0.134
**Site of infection**					0.032			
*Genital*	1230	(86.44)	1			1		
*Anorectal*	168	(11.81)	1.05	(0.94; 1.17)		1.12	(0.93; 1.34)	0.238
*Pharingeal*	25	(1.76)	0.61	(0.27; 1.38)		0.48	(0.21; 1.12)	0.089
**HIV status**					0.372			
*Negative*	1244	(86.81)	1			NI		
*Positive*	189	(13.19)	1.09	(0.90; 1.31)				
**Co-infection**^**a**^					0.001			
*No*	967	(86.65)	1			1		
*Yes*	149	(13.35)	0.65	(0.44; 0.97)		0.58	(0.38; 0.89)	0.014
**Previously diagnosed**^**b**^					0.073			
*No*	1000	(83.40)	1			1		
*Yes*	199	(16.60)	1.81	(1.24; 2.66)		2.00	(1.36; 2.96)	<0.001

Ordered logistic model for resistance categories: (AZM-R/CFM-R *vs* others, AZM-R/CFM-R and AZM-I/CFM-R *vs* others, AZM-R/CFM-R, AZM-I/CFM-R and AZM-R/CFM-S or AZM-S/CFM-R *vs* others, AZM-R/CFM-R, AZM-I/CFM-R, AZM-R/CFM-S or AZM-S/CFM-R and AZM-I/CFM-S *vs* others).

^a^ 317 data were missing

^b^ 234 data were missing; NI: variables not included in the final model because—not significant at the univariate/multivariate analysis.

^c^ univariate and multivariate logistic regressions were carried out on a complete dataset obtained by multiple imputation.

At the multivariate analysis, the MSW and women had proportional aOR respectively of 1.25 (95%CI: 0.90; 1.73) and 1.67 (95%CI: 1.16; 2.40) than MSM, for any level of resistance (see Table 2). For gonococci isolated from subjects of Eastern European (proportional aOR: 0.52; 95%CI: 0.31; 0.87), Asian (proportional aOR: 0.81; 95%CI: 0.68; 0.98) and Latin American (proportional aOR: 0.72; 95%CI: 0.46; 1.12) origins, the odds of being below given level of severity decreased from 48% to 19%. Conversely, gonococci collected from African patients had an aOR of 1.38 (95%CI: 0.90; 2.11) than Italians. Compared to the genital site, isolates collected from the pharynx had an aOR of resistance of 0.48 (95%CI: 0.21; 1.12). The proportional aOR of resistance was 0.58 (95%CI: 0.38; 0.89) for presence *vs* absence of co-infection and 2.00 (95%CI: 1.36; 2.96) for previous history *vs* no history of gonorrhoea.

### Association between patient characteristics and treatments used

In the period 2009–2016, the most frequent treatment regimens prescribed were CFM (2009–2012: N = 434 and 2013–2016: N = 165) and CRO (2009–2012: N = 189 and 2013–2016: N = 145) as monotherapy or in association with AZM (2009–2012: N = 10 and 2013–2016: N = 414).

Throughout the entire study period, 12 patients were treated with AZM alone or in combination with antimicrobials other than CRO (*e*. *g*. levofloxacin).

Table 3 reports results from univariate and multivariate analyses. Between 2009 to 2012, at both the univariate and multivariate analyses, patients with pharyngeal and anorectal site infections were, respectively, more and less likely to receive inappropriate treatment than those with infections at the genital site [anorectal site aOR of 0.27 (95%CI: 0.04; 1.70) and pharyngeal site aOR of 9.33 (95%CI: 3.25; 26.79)]. The MSW and women were more likely to receive therapy discordant with the guidelines in force than the MSM at the univariate but not at the multivariate analyses.

**Table 3:** Results from the NG antimicrobial resistance surveillance in Italy (2009–2016). Demographic and clinical characteristics of 1,433 subjects for which clinical information (antibiotic regimen, diagnosis) was available by therapy agreement (guidelines based *vs* effective therapy).

	2009–2011								2012–2014							
	*All*		*Discordant with guidelines*						*All*		*Discordant with guidelines*					
	N	(%)	N	(%)	p^c^	aOR^d^	(95%CI)	p	N	(%)	N	(%)	p^c^	aOR^d^	(95%CI)	p
**Sexual orientation and gender**					0.003	NI							<0.001			
*MSM*	399	(60.18)	13	(3.26)					479	(62.53)	108	(22.55)		1		
*MSW*	239	(36.05)	13	(5.44)					267	(34.86)	87	(32.58)		2.95	(2.36; 3.69)	<0.001
*Women*	25	(3.77)	3	(12.00)					20	(2.61)	10	(50.00)		5.26	(1.48; 18.71)	0.010
**Age (years)**					0.864	NI							0.015			
*≤ 25*	176	(26.43)	8	(4.55)					165	(21.51)	36	(21.82)		1		
*>25*	490	(73.57)	21	(4.29)					602	(78.49)	170	(28.24)		1.51	(1.08; 2.10)	0.014
**Citizenship**^**c**^					0.540	NI							<0.001			
*Italian*	535	(80.45)	25	(4.67)					672	(87.73)	176	(26.19)		1		
*Asian*	13	(1.95)	0	(0.00)					7	(0.91)	1	(14.29)		0.62	(0.38; 1.03)	0.067
*East European*	47	(7.07)	3	(6.38)					38	(4.96)	17	(44.74)		1.59	(1.21; 2.08)	0.001
*Latin American*	31	(4.66)	0	(0.00)					16	(2.09)	4	(25.00)		1.11	(0.63; 1.95)	0.722
*African*	39	(5.86)	0	(0.00)					33	(4.31)	8	(24.24)		0.74	(0.42; 1.33)	0.317
**Site of infection**					<0.001								0.139			
*Genital*	569	(86.21)	25	(4.39)		1			661	(86.63)	159	(24.05)		1		
*Anorectal*	81	(12.27)	1	(1.23)		0.27	(0.04; 1.70)	0.164	87	(11.40)	39	(44.83)		3.08	(1.20; 7.90)	0.019
*Pharyngeal*	10	(1.52)	3	(30.00)		9.33	(3.25; 26.79)	<0.001	15	(1.97)	7	(46.67)		4.61	(2.05; 10.38)	<0.001
**HIV status**					0.735	NI							0.864	NI		
*Negative*	581	(87.24)	26	(4.48)					663	(86.44)	177	(26.70)				
*Positive*	85	(12.76)	3	(3.53)					104	(13.56)	29	(27.88)				
**Co-infection**^**a**^					0.717	NI							0.089			
*No*	339	(85.61)	20	(5.90)					628	(87.22)	165	(26.27)		1		
*Yes*	57	(14.39)	4	(7.02)					92	(12.78)	38	(41.30)		1.98	(1.09; 3.61)	0.026
**Previously diagnosed**^**b**^					0.700	NI							0.035			
*No*	347	(78.15)	19	(5.48)					653	(86.49)	155	(23.74)		1		
*Yes*	97	(21.85)	4	(4.12)					102	(13.51)	50	(49.02)		3.58	(1.55; 8.29)	0.003

^a^ 317 data were missing

^b^ 234 data were missing

^c^ p values from univariate logistic regression models with cluster adjusted standard errors

^d^ univariate and multivariate logistic regressions were carried out on a complete dataset obtained by multiple imputation. NI: variables not included in the final model because—not significant at the univariate/multivariate analysis

Between 2013 and 2016, results from the univariate and multivariate analyses showed that, MSW (MSW:32.58% *vs* MSM:22.55%; aOR: 2.95; 95%CI: 2.36; 3.69; p<0.001), women (women:50.00% *vs* MSM: 22.55%; aOR: 5.26; 95%CI: 1.48; 18.71; p = 0.010), older subjects (>25 years: 28.24% *vs* ≤25 years: 21.82%; aOR: 1.51; 95%CI: 1.08; 2.10; p = 0.014), East Europeans (East Europeans: 44.74% *vs* Italians: 26.19%; aOR: 1.59; 95%CI: 1.21; 2.08; p = 0.001), subjects with pharyngeal infection (pharyngeal site: 46.67% *vs* genital site: 24.05%; aOR: 4.61; 95%CI: 2.05; 10.38; p<0.001) or anorectal infection (anorectal site: 44.83% *vs* genital site: 24.05%; aOR: 3.08; 95%CI: 1.20; 7.90; p<0.019), the co-infected (co-infected: 41.30% *vs* not co-infected: 26.27%; aOR: 1.98; 95%CI: 1.09; 3.61; p = 0.026) and those with a past history of gonorrhoea (previously diagnosed: 49.02% *vs* not previously diagnosed 23.74%; aOR: 3.58; 95%CI: 1.55; 8.29; p = 0.003) were significantly more likely to receive treatment discordant with the guidelines compared to their respective reference categories.

## Discussion

In our previous article, reporting data on gonorrhoea in Italy, a high level of AZM-R between 2007–2008 [17] and a high percentage of CFM-R in 2009 [16] were observed. In the current analysis, which refers to the period 2009–2016, a shift in antimicrobial susceptibility was observed following the endorsement of the recommended revised protocol for gonorrhoea treatment, which suggests the use of a combined therapy with CRO plus AZM in clinical practice instead of a monotherapy with ESCs, as previously recommended [9].

When analyzing the temporal trend of AZM-R, we observed that the proportion of resistant gonococci decreased until 2012, time when the new guidelines were introduced in Europe, but increased afterwards as reported in other European countries [13, 14]. Since 2012, isolates with intermediate MIC values for AZM also increased (2013: 31.18%) but decreased in the following years (2016: 6.97%) and four isolates with high levels of AZM-R (MIC≥ 256 mg/L) were identified, in line with what described elsewhere [11, 22–26].

In the same way, the proportion of CFM-R gonococci decreased from 2009 to 2011, remained fairly stable until 2014, as documented in other European countries [13] and in Canada [27], and decreased further in 2015, when CFM-R was not even detected, and in 2016 (1.39%).

As also described in Europe [28], resistant gonococci were isolated more frequently from subjects older than 25 years than in their younger counterparts, however results were no longer significant at the multivariate analyses. In particular, the percentages of AZM-I gonococci (independently from CFM resistance levels) and resistant to both (AZM and CFM) were higher in older than in younger subjects, the reverse was found for gonococci resistant to only one antimicrobial.

As reported in the Euro-GASP analysis [13, 28], having had gonorrhoea in the past increased the risk of being infected by resistant isolates while having a co-infection diminished it. Most co-infections were due to *C*. *trachomatis*, which based on the protocol set out in the guidelines, should be treated with AZM, administered as monotherapy [9]. However, in our study we observed that most of the patients with co-infections were not treated with AZM and perhaps for these reasons had lower levels of resistance.

The Euro-GASP study [28] found a high level of resistance to CFM and AZM among MSW and women compared to MSM, in 2013, but, one year later, observed an increase of AZM resistance levels in MSM and a decrease of it in women [13]. A surveillance programme, carried out in England and in Wales between 2007–2011, also reported a strong association between isolates with decreased susceptibility to CFM and gonorrhoea infection in MSM [29]. Similarly, a surveillance programme carried out in 30 US cities, between 2005 and 2010, found higher rates of infections due to resistant gonococci among MSM than other patients [30].

In our study, the 61.44% of gonorrhoea infections and the 95.83% of anorectal infections, were found among MSM. However, the probability of being infected by CFM-R and/or AZM-I/AZM-R isolates, was slightly higher in MSW than in MSM.

Overall, the combined therapy for gonorrhoea was always prescribed as suggested. However, CFM as monotherapy was prescribed also after 2013 when the guidelines did not longer support its use, except for some specific conditions (*e*.*g*. when an intramuscular injection is not possible or refused by a patient) [9]. In this regard, we found that the 37% of MSW patients received, as monotherapy treatment, CFM or CRO after 2013 compared to the 19% of the MSM, and, that the MSW were less likely than MSM to be treated in accordance with the guidelines. However, the percentage of susceptibility to CFM was 97% among MSW, still above the recommended 95% treatment threshold, set by the World Health Organisation (WHO), and no CRO resistant gonococci have been found [31].

In addition, the older subjects, those with an anorectal or pharyngeal infection, the co-infected subjects and those with a previous history of gonorrhoea infection, at least for the period 2013–2016, were not always correctly treated. But, since these could represent the most serious cases, therapies may be difficult to assess without having extensive information on treatment regimens (antimicrobial or other drugs) and on other diseases they might have.

Some limits of the study can be highlighted: 1) our data about antimicrobial prescriptions come from laboratories participating in a network and therefore our results cannot be generalized to the whole country; 2) missing records for the variables co-infection and history of gonorrhoea comprised, respectively, 24.50% and 19.85% of all records. However, all statistical models were fitted to data with forty rounds of multiple imputation to minimize bias due to missing data.

In conclusion, our findings demonstrate that shifts in CFM and AZM susceptibility in *N*. *gonorrhoeae* have occurred in Italy over the study period. The increasing rates of resistance against AZM in 2015 and 2016, suggest that the use of this drug in the current treatment scheme is still correct but it is necessary to monitor the AZM susceptibility trend over time.

In many countries [13, 14, 16, 27, 29], the percentages of AZM and CFM resistance have declined over the years and this decline coincided with the introduction of the combined therapy in clinical practice. However, resistance to CFM is still a concern, even if gonococci with decreased susceptibility to this antimicrobial *in vitro* appear to be susceptible to other antimicrobials, such as CRO, as also found in this study. Globally, gonococci susceptible to CRO are the majority; but, AZM-R gonococci are prevalent worldwide and the concomitant resistance to CRO and AZM has been also documented [3, 4, 6].

For the first time, patient characteristics predictive of being infected by *N*. *gonorrhoeae* resistant isolates were identified; moreover, the current therapy for gonorrhoea *vs* the implementation of therapy scheme as in the guidelines was also evaluated. For all the above mentioned reasons, gonorrhoea treatment strategy should be based on the evidence obtained by the local antimicrobial surveillance system and on data about treatment failures [32].

## Materials and methods

### *Neisseria gonorrhoeae* isolates and antimicrobial susceptibility

Data on NG antimicrobial resistance in Italy were obtained from a network coordinated by the Istituto Superiore di Sanità (ISS) with the support of the Italian Ministry of Health. Sexually transmitted diseases clinics and other laboratories in 12 cities throughout the country (6 in the North, 3 in the Centre of Italy and 3 in the South for a total of 14 collaborating laboratories) participated in the project [33]. Primary isolation, identification, and collection of gonococci, following standard microbiological procedures, were completed by the collaborating laboratories. Briefly, isolates stored at −80°C in brain heart infusion medium (Oxoid, Ltd, Italy) containing 20% glycerol were sent bimonthly to ISS after 18 to 24 h of growth at 37°C in a 5% CO_2_ atmosphere on Thayer-Martin agar plates (Oxoid Ltd, Italy) with 1% IsoVitalex (Oxoid Ltd, Italy).

Unlinked anonymous demographic, clinical, and laboratory data were received and recorded at the ISS, using Epi-Info software (version 3.3.2, 2005). Prescribed therapies were obtained, when available, from each collaborating center by filling a form with the type of antimicrobial used.

Following the Euro-GASP scheme [34], isolates were tested by ISS for antimicrobial susceptibility to CFM, AZM and CRO after growth on Thayer-Martin medium (Oxoid Ltd, Italy) with 1% IsoVitalex (Oxoid Ltd, Italy) at 37°C in a 5% CO_2_ atmosphere. The Etest (bioMérieux, Sweden) and MIC TEST STRIP methods (Liofilchem Diagnostici, Italy) were carried out following standard procedures. Breakpoints were those indicated by EUCAST (version 7.1, 2017) [35]: for CFM and CRO, susceptible with MIC values ≤0.125 mg/L and resistant with MIC values > 0.125 mg/L; for AZM, susceptible with MIC values ≤0.25, resistant with MIC values > 0.5 mg/L and intermediate isolates with MIC values of 0.5 and 0.38 mg/L. The World Health Organization (WHO) *N*. *gonorrhoeae* G, K, M, O, and P control strains were used in each assay [36].

### Statistical analysis

From 2009 to 2016, laboratories from local hospitals confirmed 1,937 gonorrhoea cases by culture and the isolates were sent to ISS. Out of 1,937, a total of 1,688 gonococci were examined for antimicrobial susceptibility to AZM, CFM and CRO, 270 records with missing information about therapy were excluded leaving a total of 1,433 isolates.

Considering the antimicrobial susceptibility categories for AZM and CFM, gonococci were divided into five categories: “susceptible to AZM and CFM”, “intermediate to AZM and susceptible to CFM”, “resistant to AZM and susceptible to CFM”, “susceptible or intermediate to AZM and resistant to CFM” and “resistant to AZM and resistant to CFM”. Patient’s age was split in two groups: < 25 years, 25 years and older. Gender and sexual orientations were coded as: MSM, MSW and women. Co-infection was defined according to the presence or absence of a co-infection with *Chlamydia trachomatis* and/or *Treponema pallidum* and/or *Trichomonas vaginalis*.

Ordered logit regressions were carried out to investigate the association of resistance severity levels with demographic and clinical patient characteristics. Our initial models included all variables which were statistically significant at the univariate analysis (p <0.2). Then we used a step approach, dropping at each round not significant variables (p> = 0.2).We calculated p values with the Wald test on combined OR and SE estimates by application of Rubin's rules for combining multiply imputed data. Cluster robust standard errors were used to take into account intra laboratories correlations.

Recommended treatments for gonorrhoea were extrapolated from the published 2009 [8] and 2012 [9] guidelines. For each antimicrobials prescribed, the discrepancy with the guidelines was evaluated. Logistic regressions with cluster robust standard errors were carried out to investigate the association with patients demographic (age, sex, sexual orientation, year of occurrence) and clinical (site of infection, diagnosis, history of gonorrhoea, presence of co-infection, HIV status) characteristics. Considering the guidelines published during the study period, the analysis was carried out separately for the years 2009–2012 and 2013–2016. To minimize bias due to missing data, all statistical models were fitted to data with forty rounds of multiple imputation, using chained equations [37], with all terms contained within the imputation model. All analyses were carried out in STATA 13.1 [38].
